# Computed Tomography Registration-Derived Regional Ventilation Indices Compared to Global Lung Function Parameters in Patients With COPD

**DOI:** 10.3389/fphys.2022.862186

**Published:** 2022-05-26

**Authors:** Julien Cohen, Mehdi Shekarnabi, Marie Destors, Renaud Tamisier, Sandrine Bouzon, Maciej Orkisz, Gilbert R. Ferretti, Jean-Louis Pépin, Sam Bayat

**Affiliations:** ^1^ Department of Radiology, Grenoble University Hospital, Grenoble, France; ^2^ Department of Imaging, Neuchatel Hospital Network (RHNE), Neuchatel, Switzerland; ^3^ Inserm UA07 STROBE Laboratory, Université Grenoble Alpes, Grenoble, France; ^4^ INSA-Lyon, CNRS, Inserm, CREATIS UMR 5220, University Lyon, Université Claude Bernard Lyon 1, Lyon, France; ^5^ HP2 Laboratory, Inserm U1300, Université Grenoble Alpes, Grenoble, France; ^6^ Department of Pulmonology and Physiology, Grenoble University Hospital, Grenoble, France

**Keywords:** chronic obstructive pulmonary disease, computed tomography, lung function tests, image processing, regional lung ventilation

## Abstract

CT registration-derived indices provide data on regional lung functional changes in COPD. However, because unlike spirometry which involves dynamic maximal breathing maneuvers, CT-based functional parameters are assessed between two static breath-holds, it is not clear how regional and global lung function parameters relate to each other. We assessed the relationship between CT-density change (dHU), specific volume change (dsV), and regional lung tissue deformation (J) with global spirometric and plethysmographic parameters, gas exchange, exercise capacity, dyspnoea, and disease stage in a prospective cohort study in 102 COPD patients. There were positive correlations of dHU, dsV, and J with spirometric variables, DLCO and gas exchange, 6-min walking distance, and negative correlations with plethysmographic lung volumes and indices of trapping and lung distension as well as GOLD stage. Stepwise regression identified FEV1/FVC (standardized *β* = 0.429, *p* < 0.0001), RV/TLC (*β* = −0.37, *p* < 0.0001), and BMI (*β* = 0.27, *p*=<0.001) as the strongest predictors of CT intensity-based metrics dHU, with similar findings for dsV, while FEV1/FVC (*β* = 0.32, *p*=<0.001) and RV/TLC (*β* = −0.48, *p*=<0.0001) were identified as those for J. These data suggest that regional lung function is related to two major pathophysiological processes involved in global lung function deterioration in COPD: chronic airflow obstruction and gas trapping, with an additional contribution of nutritional status, which in turn determines respiratory muscle strength. Our data confirm previous findings in the literature, suggesting the potential of CT image-based regional lung function metrics as the biomarkers of disease severity and provide mechanistic insight into the interpretation of regional lung function indices in patients with COPD.

## Introduction

COPD is increasingly recognized as a complex disease with diverse trajectories in disease progression (Han et al., 2010; Agustí et al., 2017). Indeed, there is significant heterogeneity in clinical features, physiology, lung parenchymal and airway morphology, response to therapy, the decline in lung function, and survival in COPD patients (Lange et al., 2016). The diagnosis and assessment of COPD severity are mainly based on the detection of chronic flow limitation on post-bronchodilator spirometry (Han et al., 2010). However, there is consensus that basic spirometric parameters such as FEV1 do not adequately describe disease heterogeneity and are insufficient for the optimal assessment and management of COPD. Novel means of identifying clinical traits or phenotypes that may have consequences on the management and clinical outcome in COPD patients are needed (Van Der Molen et al., 2013; Garudadri and Woodruff, 2018; Segal and Martinez, 2018; Manian, 2019; Martin et al., 2019; Burkes et al., 2021).

Computed tomography has been extensively used for phenotyping COPD by characterizing airway and parenchymal morphology (Washko et al., 2012; Kauczor et al., 2019). One of the emerging applications of CT is the measurement of regional functional indices based on the matching or registration of static images acquired at total lung capacity (TLC) and residual volume (RV) or functional residual capacity (FRC). In contrast to spirometry, which is a global measure of the respiratory system, CT image registration-based functional indices provide data on the spatial distribution of functional changes, and there is increasing evidence that regional functional changes can precede the detection of disease by spirometry (Bodduluri et al., 2018).

Image registration allows deforming a lung image acquired at a given volume to match the shape of that acquired at a different level of lung inflation. This allows the voxel-to-voxel assessment of lung density change, which reflects the local change in air volume (Ding et al., 2012). This measurement has been shown to closely correlate with regional lung ventilation (Reinhardt et al., 2008). An alternative approach is to measure the amount of regional deformation, which reflects the local tissue biomechanical properties and strain (Ding et al., 2012).

There are, however, fundamental differences between CT registration-based and global spirometric measurements of lung functions. Because spirometry involves dynamic, forced maximal breathing maneuvers, parameters such as FEV1 or expiratory flows are determined by not only the subject’s lung volume, respiratory muscle strength, and airway resistance, but also by the elastic lung recoil and airway wall elastance (Macklem and Mead, 1967). CT-based functional parameters, on the other hand, are assessed between two static breath-hold conditions. This raises the question of how the regional CT-based functional parameters and global lung function parameters relate to each other.

This study aimed at assessing the relationship between CT registration-based regional lung function parameters, namely CT-density change, specific volume change, and regional lung tissue deformation in comparison with global spirometric and plethysmographic parameters, in COPD patients. We further extended this comparison to diffusion capacity, gas exchange, exercise capacity, dyspnoea, and disease stage. Specifically, we aimed at determining which global lung function parameter or combination of parameters relates to regional lung function indices.

## Materials and Methods

### Ethics and Consent

Patients included in this study were part of two prospective COPD cohorts of Grenoble University Hospital (Grenoble, France), registered with ClinicalTrials.gov (NCT00404430 and NCT03014609). The studies were approved by an independent ethics committee. The retrospective analysis of CT images in this ancillary study was approved by the Comité de Protection des Personnes, CHU Grenoble - approval 2016-A01657-44 and 2006-A00491-50/4. All participants gave written informed consent to participate in the study.

### Subjects

Patients aged >18 years with COPD, followed up at the Grenoble University Hospital outpatient pulmonology clinic were included in the cohorts. Patients with evolving cancer; heart failure with an ejection fraction <45% or pregnancy were not included. Overall, 121 and 77 patients were included in the two cohorts, respectively. Patients with available CT imaging at both TLC and RV as well as forced spirometry at the initial visit (*n* = 102) were included in the study.

### CT Imaging and Image Processing

Chest CT was performed at the Grenoble University Hospital Department of Radiology, with a 256-slice scanner (GE Revolution CT, GE Medical Systems, Milwaukee, United States) with the following settings: 120 kV, tube current modulation, and collimation width of 0.625 or 0.4 mm. The patients were instructed by the technician prior to and coached during image acquisition. Images were reconstructed with a standard convolution kernel and a matrix size of 512 × 512 pixels.

Details of the image processing are included in the online supplement. Briefly, after an initial rigid alignment of the inspiratory and expiratory volumes, the lung was separated from surrounding structures by image segmentation. Deformable image registration was then applied to warp the inspiration image to morphologically match the expiration image (Heinrich et al., 2013).

The following outcome measures were computed:

1) the inspiratory—expiratory x-ray density changes in Hounsfield units (*dHU*) between the fixed (expiration) and warped (inspiration) images. Voxels within the −1,100 to −500 HU were included in the analysis, excluding denser tissue structures such as blood vessels.

2) The specific volume change (*dsV*) between fixed and warped images, as defined by Simon et al. (Simon, 2000), which is the difference between the inspiration and expiration volumes normalized to the expiratory volume.

3) The determinant of the Jacobian matrix, referred to as the Jacobian (*J*), which expresses the local relative volume change from inspiration to expiration (Fleming, 2012). The Jacobian is independent of the image intensity values and therefore of *dHU*. A *J* > 1 means local expansion whereas a *J* < 1 indicates local contraction and *J* = 1 indicates no volume change.

Scattering in dHU, dsV, and J was expressed as the quantile variation coefficient (QVC) defined as interquartile range/median rather than coefficient of variation, given the non-normal distribution of the parameters.

### Global Lung Function and Exercise Capacity

Spirometry, body plethysmography, and carbon monoxide diffusion capacity (DLCO) measurements were performed using a Medisoft body box (MGC Diagnostics, MN, United States), in accordance with the American Thoracic Society–European Respiratory Society technical recommendations (Graham et al., 2019). Post-bronchodilator spirometric parameters expressed with reference to the Global Lung Initiative reference values (Quanjer et al., 2012) were used in the analyses. Blood gas and resting oxygen saturation measurements were performed using a Siemens RAPIDPoint 500 blood gas analyzer (Siemens Healthcare SAS, Saint-Denis, France). Six-minute walking tests were performed following the ATS guidelines (Laboratories, 2002). Dyspnoea was assessed using the Borg scale (Borg, 1982).

### Data Analysis

Data are presented as median, interquartile range (Q1–Q3). Pearson correlation was used to assess the relationship between the metrics of the regional lung function and overall lung function parameters, gas exchange and exercise capacity, and potential effect-modifying variables such as smoking history, age, sex, and BMI. A *p* < 0.05 was considered significant for all tests. Variables with significant associations with regional lung function in the univariate analysis were included in a forward stepwise multiple linear regression model. Multicollinearity diagnostics were performed, and variables with a variance inflation factor (VIF) greater than 10 were excluded from the model. Variables with non-normal distribution based on a Shapiro test were square-root transformed (BMI, dHU, dsV, *J*). Skewness and kurtosis of the parameter distributions were assessed on nontransformed parameters. All statistical analyses were performed using R statistical software (Version 1.4.1106, R Foundation for Statistical Computing, Vienna, Austria; http://www.R-project.org).

## Results

Subject characteristics are shown in Table 1. A total of 102 patients fulfilling the inclusion criteria were included in the study. All GOLD stages were represented with the majority of patients in GOLD 2 and 3 stages. Table 2 shows the lung volume and regional lung function parameters, as well as the scattering and indices describing the shape of their respective distributions, kurtosis, and skewness. Scattering in the Jacobian was relatively smaller than that of dsV and dHU.

**TABLE 1:** Subject characteristics.

	Med (IQR)
Age years	65.3 (58.9,71.1)
Sex M/F	72/30
BMI kg·m^−2^	26.1 (22.3,29.7)
Smoking pack-years	40.3 (29.3,51.8)
FVC %pred	77.7 (68.4,89)
FVC z-score	−1.5 (−2.1,−0.7)
FEV1 %pred	54.3 (42,64.6)
FEV1 z-score	−2.8 (−3.6,−2.1)
FEV1/FVC %	53.4 (46.6,63.9)
FEV1/FVC z-score	−2.7 (−3.5,−1.9)
FEF25-75 %pred	43.1 (23.8,60.8)
TLC %pred	116.1 (106.1,132.4)
RV %pred	176.1 (141.2,202.9)
RV/TLC %	56.1 (49.8,60.9)
DLCOcorr %pred	57.1 (31.7,68.5)
PaO_2_ kPa	10 (9.1,10.8)
PaCO_2_ kPa	4.9 (4.6,5.2)
SaO_2_%	95 (94,96)
6MWD m	485 (382,550)
Dyspnoea score	0.5 (0,2)
GOLD 1 n (%)	9 (8.8)
GOLD 2 n (%)	53 (52.0)
GOLD 3 n (%)	28 (27.5)
GOLD 4 n (%)	12 (11.8)

Data are median (interquartile range). BMI, body mass index; FEV1, forced expiratory volume in 1 s; FVC, forced vital capacity; FEF25-75, forced mid-expiratory flow; RV, plethysmographic residual volume; TLC, plethsymographic total lung capacity; DLCOcorr, diffusing capacity or carbon monoxide, corrected for hemoglobin value; PaO_2_, arterial O_2_ pressure; PaCO_2_, arterial CO_2_ pressure; SaO_2_, arterial O_2_ saturation; 6MWD, 6-min walk distance; GOLD, Global Initiative for Chronic Obstructive Lung Disease class. Smoking history was available in 98 subjects, blood gases, exercise capacity data in 95, and diffusion capacity in 58.

**TABLE 2:** Image registration-derived parameters.

	Med (IQR)
V_insp_ L	5.75 (4.94,6.6)
V_exp_ L	3.94 (3.24,4.57)
dV L	1.88 (1.34,2.34)
dHU (HU)	56.5 (39.6,76.0)
dHU-QVC	0.80 (0.8,1.0)
dHU-kurtosis	9.1 (6.8,12.4)
dHU-skewness	1.0 (0.7,1.4)
dsV	0.69 (0.46,0.88)
dsV-QVC	0.90 (0.81,1.04)
dsV-kurtosis	23.08 (15.71,35.97)
dsV-skewness	2.92 (2.37,3.69)
J	1.41 (1.31,1.54)
J-QVC	0.22 (0.16,0.28)
J-kurtosis	14.92 (10.55,21.04)
J-skewness	2.02 (1.47,2.66)

Data are median (interquartile range). *V*_insp_, inspiratory lung image volume; *V*_exp_, expiratory lung image volume; dV, inspiration—expiration volume change; dHU, expiration—inspiration lung density change; QVC, quantile variation coefficient; dHU-kurtosis, density change distribution kurtosis; dsV, inspiration—expiration specific volume change; dsV-kurtosis, specific volume change distribution kurtosis; *J*, Jacobian determinant expiration → inspiration; J-kurtosis, Jacobian determinant distribution kurtosis.

**FIGURE 1:**
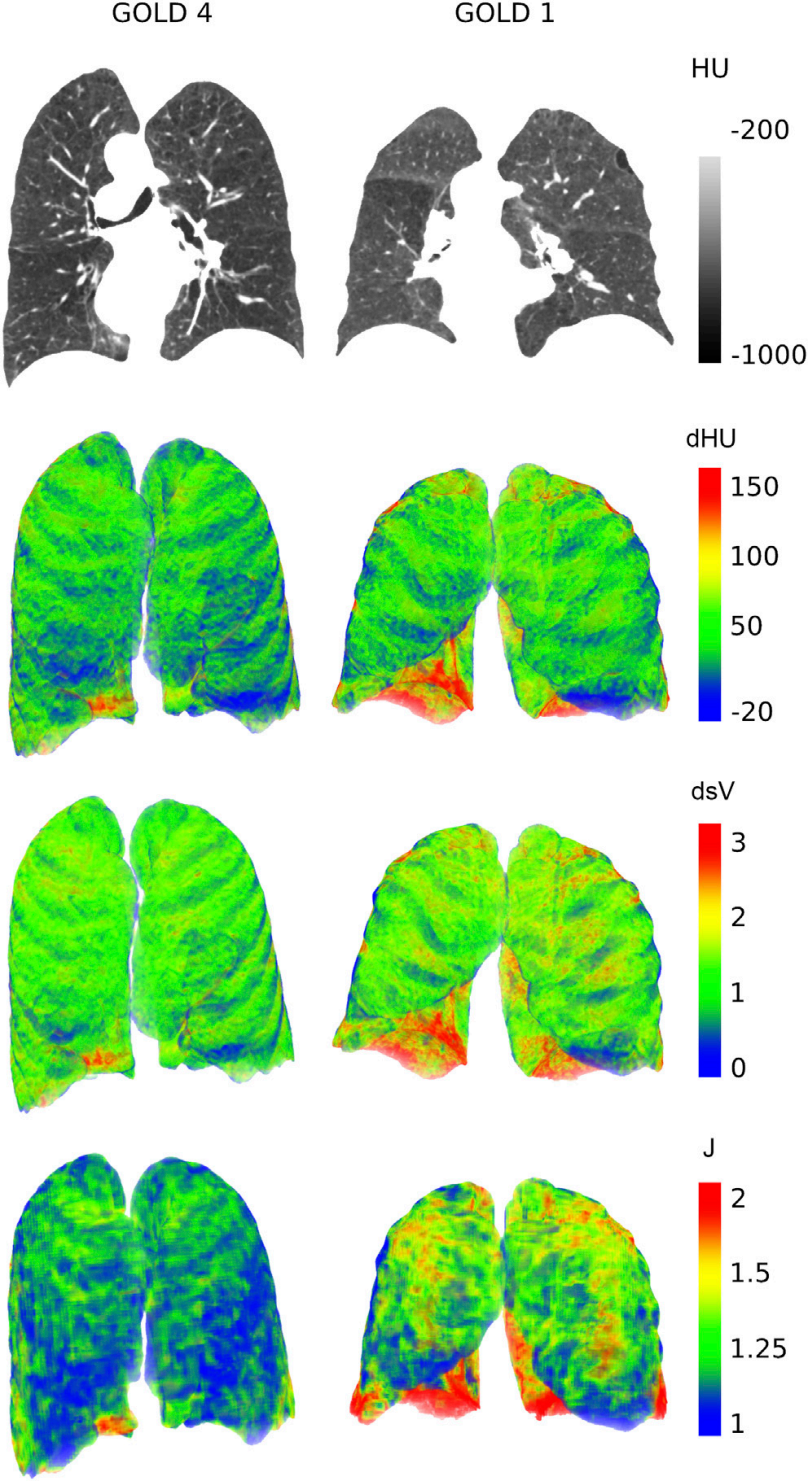
Coronal expiratory CT slices (Top) and 3D renderings of the regional density change (dHU), specific volume change (dsV), and Jacobian determinant (*J*) in a representative GOLD-1 and GOLD-4 patient. Note the remarkably lower local values of dHU, dsV, and *J* in the Gold-4 patients compared to the Gold-1 patient.

Figure 1 shows examples of raw CT images and 3D-rendered maps of the regional lung function parameters in 2 representative GOLD 1 and 4 patients. Correlations between regional lung function parameters and demographic, global lung function, gas exchange, exercise capacity, dyspnea, and GOLD classification data are shown in Table 3. The Jacobian, dHU, and dsV were significantly and positively related to BMI but not to age, sex, or smoking history. All parameters showed positive correlations with FEV1, FEV1/FVC, and FEF25-75. Conversely, significant inverse correlations were found with indices of lung hyperinflation, TLC, RV, and RV/TLC. All regional lung function parameters showed positive correlations with resting PaO_2_, oxygen saturation and 6-min walking distance but negative correlations with GOLD stage. The Jacobian was inversely correlated with the resting dyspnoea score.

**TABLE 3:** Correlation between the regional lung function parameters and demographic, global lung function, gas exchange, and exercise capacity, dyspnoea and GOLD classification data.

	*J*		*dHU*		*dsV*	
	R	*p*	R	*p*	R	*p*
Age yrs	−0.07	0.456	−0.14	0.154	−0.10	0.301
Sex M/F	−0.12	0.226	−0.09	0.380	−0.05	0.610
BMI kg·m^−2^	0.24	0.016	0.34	<0.0001	0.25	0.013
Smoking pack-yrs	−0.03	0.749	−0.01	0.896	0.01	0.919
FVC %pred	0.28	0.004	0.20	0.050	0.28	0.004
FEV1 %pred	0.50	<0.0001	0.51	<0.0001	0.51	<0.0001
FEV1/FVC %	0.53	<0.0001	0.62	<0.0001	0.54	<0.0001
FEF25-75 %pred	0.51	<0.0001	0.55	<0.0001	0.53	<0.0001
TLC %pred	−0.42	<0.0001	−0.47	<0.0001	−0.43	<0.0001
RV %pred	−0.52	<0.0001	−0.50	<0.0001	−0.51	<0.0001
RV/TLC %	−0.52	<0.0001	−0.46	<0.0001	−0.51	<0.0001
DLCOcorr %pred	0.36	0.005	0.42	0.001	0.42	0.001
PaO_2_ kPa	0.35	0.001	0.35	0.001	0.38	<0.0001
PaCO_2_ kPa	−0.11	0.292	−0.10	0.330	−0.10	0.332
SaO_2_%	0.28	0.006	0.30	0.004	0.30	0.004
6MWD	0.29	0.004	0.32	0.001	0.37	<0.0001
Dyspneoa score	−0.30	0.029	−0.20	0.147	−0.27	0.052
GOLD	−0.50	<0.0001	−0.44	<0.0001	−0.47	<0.0001

dHU, expiration—inspiration lung density change; dsV, inspiration—expiration specific volume change; J, Jacobian determinant expiration → inspiration; R, Spearman correlation coefficient; M/F, male/female; BMI, body mass index; FEV1, forced expiratory volume in one second; FVC, forced vital capacity; FEF25-75, forced mid-expiratory flow; RV, plethysmographic residual volume; TLC, plethsymographic total lung capacity; DLCOcorr; diffusing capacity or carbon monoxide, corrected for hemoglobin value; PaO_2_, arterial O_2_ pressure; PaCO_2_, arterial CO_2_ pressure; SaO_2_, arterial O_2_ saturation; 6MWD, 6-min walk distance; GOLD, Global Initiative for Chronic Obstructive Lung Disease class.

Table 4 shows the results of forward multiple linear regression analysis. This analysis indicated that both dHU and dsV could be predicted from a combination of FEV1/FVC, RV/TLC, and BMI. Regarding the Jacobian, variation in this parameter was described by the combination of FEV1/FVC and RV/TLC.

**TABLE 4:** Stepwise multiple regression parameter estimates.

		Unstandardized		Standardized	
		*β*	*SE*	*β*	*p*
*dHU*					
	FEV1/FVC	0.076	0.013	0.429	0.0000
	RV/TLC	−0.084	0.017	−0.371	0.0000
	BMI	1.059	0.273	0.272	0.0002
*dsV*					
	FEV1/FVC	0.006	0.001	0.327	0.0002
	RV/TLC	−0.01	0.002	−0.444	0.0000
	BMI	0.076	0.03	0.195	0.0126
*J*					
	FEV1/FVC	2.00E-03	0	0.323	0.0002
	RV/TLC	-4.00E-03	0.001	−0.477	0.0000

dHU, expiration—inspiration lung density change; dsV, inspiration—expiration specific volume change; *J*, Jacobian determinant expiration → inspiration; *β*, degree of change in the outcome variable for every 1-unit of change in the predictor variable; SE, standard error; standardized *β*, the fraction of total variance explained by the predictor variable.

**FIGURE 2:**
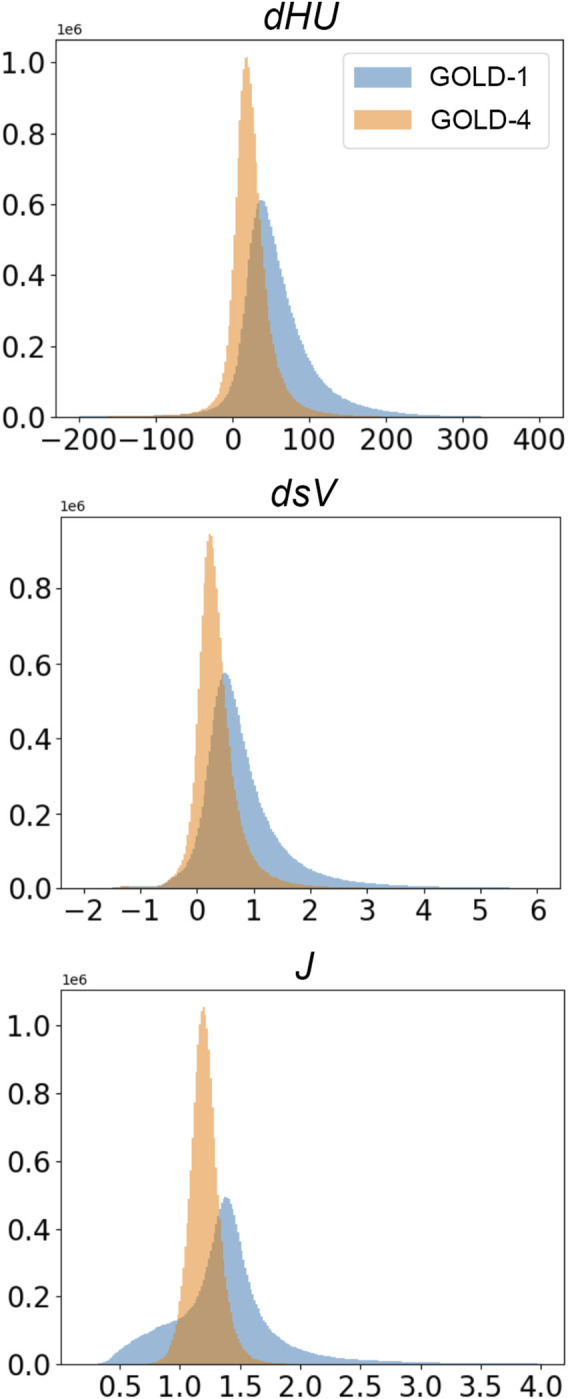
Probability distributions of the density change (dHU), specific volume change (dsV), and Jacobian determinant (J) in a representative GOLD-1 and GOLD-4 patient.

Figure 2 shows sample distributions of the regional lung function parameters in two patients, as shown in Figure 1. Table 5 shows the relation between scattering in regional lung function parameters and demographic, and global functional data. The relations between kurtosis and skewness of the regional lung function parameter distributions and global data are shown in Supplemental Tables S1,2, respectively.

**TABLE 5:** Correlation between the scattering of the regional lung function parameters assessed as quantile variation coefficient (IQR/median) and demographic, global lung function, gas exchange exercise capacity, dyspnoea, and GOLD classification data.

	*J-QVC*		*dHU-QVC*		*dsV-QVC*	
	R	*p*	R	*p*	R	*p*
Age yrs	0.06	0.577	0.08	0.447	0.10	0.300
Sex M/F	0.11	0.289	−0.09	0.375	−0.10	0.302
BMI kg·m^−2^	0.15	0.127	−0.16	0.106	−0.14	0.165
Smoking pack-yrs	−0.09	0.392	0.03	0.802	0.04	0.712
FVC %pred	0.24	0.017	−0.19	0.058	−0.17	0.082
FEV1 %pred	0.35	0.000	−0.32	0.001	−0.30	0.003
FEV1/FVC %	0.32	0.001	−0.34	0.001	−0.31	0.002
FEF25-75 %pred	0.31	0.002	−0.27	0.006	−0.25	0.012
TLC %pred	−0.13	0.195	0.25	0.015	0.21	0.046
RV %pred	−0.21	0.042	0.36	0.000	0.30	0.004
RV/TLC %	−0.27	0.009	0.40	0.000	0.37	0.000
DLCOcorr %pred	0.39	0.003	−0.34	0.008	−0.33	0.011
PaO_2_ kPa	0.28	0.006	−0.32	0.001	−0.31	0.002
PaCO_2_ kPa	−0.04	0.676	0.05	0.647	0.05	0.652
SaO_2_%	0.25	0.017	−0.27	0.010	−0.26	0.014
6MWD m	0.34	0.001	−0.30	0.003	−0.29	0.004
Dyspnoea score	−0.31	0.023	0.12	0.378	0.12	0.408
GOLD	−0.29	0.003	0.28	0.004	0.26	0.008

dHU-QVC, dHU quantile variation coefficient; dsV-CoV, QVC, specific volume change quantile variation coefficient; J-QVC, Jacobian determinant quantile variation coefficient. *R*, Spearman correlation coefficient; M/F, male/female; BMI, body mass index; FEV1, forced expiratory volume in 1 s; FVC, forced vital capacity; FEF25-75, forced mid-expiratory flow; RV, plethysmographic residual volume; TLC, plethsymographic total lung capacity; DLCOcorr; diffusing capacity or carbon monoxide, corrected for hemoglobin value; PaO_2_, arterial O_2_ pressure; PaCO_2_, arterial CO_2_ pressure; SaO_2_, arterial O_2_ saturation; 6MWD, 6-min walk distance; GOLD, Global Initiative for Chronic Obstructive Lung Disease class.

## Discussion

The main findings of this study were as follows: 1) all three regional lung function indices positively correlated to global spirometry parameters, while they were inversely correlated to plethysmographic lung volume measurements. They further correlated with PaO_2_ and exercise capacity while they were inversely correlated with dyspnoea and disease severity as reflected by the GOLD stage; 2) FEV1/FVC and RV/TLC were related to the variability in the regional lung function indices, with the additional contribution of BMI for dHU and dsV which are intensity-based indices, unlike *J* which is a biomechanical strain index; 3) the scattering in dHU and dsV, but not J, showed opposite relationships with the respective spirometry and plethysmography parameters; and 4) the frequency distribution of dsV significantly peaked (higher kurtosis) and became less asymmetric (lower skewness) with disease severity, was positively related to FEV1/FVC, and inversely to RV/TLC, DLCO, gas exchange, and exercise capacity.

In CT imaging, as the gas content within an image voxel increases, the x-ray attenuation or CT density in HU decreases. The density change dHU has therefore been taken to represent a local change in the gas volume (Gattinoni et al., 1995; Victorino et al., 2004). However, this relationship may be nonlinear and depends on the initial local density and the magnitude of density change (Simon, 2005). Here, we analyzed both dHU, and dsV which includes dHU in its calculation and is therefore not an independent outcome measure, for the sake of comparison. Overall, we found similar correlations between these two indices as well as the Jacobian, with global spirometry, plethysmography, gas exchange, and exercise capacity. Only dyspnoea score was significantly correlated to the Jacobian but not with the intensity-based indices of the regional lung function.

The stepwise regression analysis shows that the two composite parameters, FEV1/FVC and RV/TLC, explained a large part of the variance in the three regional lung function parameters. While FEV1/FVC is strongly determined by airflow limitation, hence resistance to the flow of the airway tree and lung elastic recoil, RV/TLC is determined by trapping, and lung distension. The latter parameter showed an inverse relationship with all three regional lung function indices. Our data suggest, therefore, that these two pathophysiological mechanisms, namely, airflow limitation and gas trapping are both involved in the regional lung function alteration with progressive disease severity in COPD. This can be explained by the fact that the regional lung ventilation distribution depends on the regional mechanical time constant of the lungs, which is the product of airway resistance and regional lung tissue compliance based on the analogy with an electrical circuit (Otis et al., 1956). This mechanical time constant determines the behavior of pulmonary airspaces in response to a given sinusoidal driving pressure. In COPD, both the loss of parenchymal elastic recoil and increased airway resistance may lengthen this time constant, thereby lagging volume change with respect to the pressure change under dynamic breathing conditions. Another contributing mechanism may be the presence of collateral channels whose role may be more prominent in emphysema through an increased airway resistance and a concomitantly reduced collateral resistance (Hogg et al., 1969).

We found that BMI was a significant predictor of the intensity-based regional lung function indices dHU and dsV. It is well established that many patients with COPD lose weight and become malnourished (Vandenbergh et al., 1967; Hunter et al., 1981; Sahebjami and Macgee, 1983; Braun et al., 1984; Zhang et al., 2021). Because malnutrition is associated with significant impairment of the respiratory muscle strength and endurance (Arora and Rochester, 1982), its presence in COPD may worsen the existing respiratory muscle dysfunction due to chronic airflow limitation and hyperinflation (Sahebjami and Sathianpitayakul, 2000). Our findings suggest that the regional lung function is further determined by BMI which reflects the nutritional status, a potentially important factor for the respiratory muscle function, in patients with COPD.

Previously, Bodduluri et al. found that in patients with COPD the Jacobian determinant correlated strongly with FEV1/FVC, and FEV1% predicted as well the quality of life, BODE index, and mortality on follow-up (Bodduluri et al., 2017). Although CT density-based measures of gas trapping and emphysema also correlate with airflow obstruction, there is also substantial discordance in some COPD patients (Bhatt et al., 2014). In these patients, the prediction of airflow obstruction is significantly improved by the mean Jacobian determinant (Bodduluri et al., 2017). Moreover, the appropriate CT density threshold used to differentiate emphysematous from non-emphysematous tissue is subject to the image acquisition and reconstruction parameters (Boedeker et al., 2004; Stoel et al., 2008). So far, no data are available on the comparison of intensity-based metrics of the regional lung function (dHU and dsV) and their relation to both spirometric airflow obstruction and plethysmographic air trapping and tissue distension.

We found significant changes in the frequency distribution of dHU and dSV with the COPD stage. The QVCs of dHU and dsV were inversely related to FEV1 and FEV1/FVC, DLCO, gas exchange, and 6MWD, while it was positively related to plethysmographic measures of trapping and lung distension (TLC, RV, RV/TLC) and GOLD stage. This suggests a larger relative spatial inhomogeneity in these regional lung function parameters as the disease progresses. This finding is in agreement with the study of Aliverti et al. (2013) in healthy and emphysematous subjects demonstrating a significantly higher dHU-QVC in patients with emphysema. The Jacobian QVC was, on the other hand, positively related to spirometric indices. The reason for this discrepancy is unclear. The examination of individual probability distributions reveals that the scattering in *J* decreases more than its median with disease severity (Figure 2). A potential explanation could be that a significant contribution to the scattering in *J* is due to the gravity dependence of this biomechanical index in less severe subjects. The relative contribution of gravity dependence seems to decrease with disease severity in COPD patients as suggested by the comparison of dorsal and ventral views of 3D Jacobian maps (see Supplemental Figure S1). Further study is needed, however, to assess this hypothesis.

## Conclusion

In this study, we assessed the relationship between lung CT biomechanical (Jacobian) and intensity-based (density change, specific volume change) metrics of the regional lung function with global spirometric and plethysmographic parameters, diffusion capacity, gas exchange, exercise capacity, dyspnoea, and disease stage in COPD patients. Our data show strong positive correlations with spirometric variables, DLCO, gas exchange, 6-min walking distance, and negative correlations with plethysmographic lung volumes and indices of trapping and lung distension as well as the GOLD stage. Stepwise regression identified FEV1/FVC, RV/TLC, and BMI as the strongest predictors of the variance of CT intensity-based metrics dHU and dsV, while FEV1/FVC and RV/TLC were identified as those for the Jacobian determinant. Our data suggest that the regional lung function is determined by two major pathophysiological processes involved in the global lung function deterioration in COPD, namely, chronic airflow obstruction and gas trapping, with an additional contribution of BMI, potentially through the nutritional status, which in turn determines the respiratory muscle strength. These findings confirm previous findings in the literature, suggesting the potential of CT image-based regional lung function metrics as the biomarkers of disease severity and provide mechanistic insight into the interpretation of the regional lung function indices in patients with COPD.

## Data Availability

The raw data supporting the conclusion of this article will be made available by the authors, without undue reservation.

## References

[B1] Agustí A. Celli B. Faner R. (2017). What Does Endotyping Mean for Treatment in Chronic Obstructive Pulmonary Disease? The Lancet 390, 980–987. 10.1016/s0140-6736(17)32136-0 28872030

[B2] Aliverti A. Pennati F. Salito C. Woods J. C. (2013). Regional Lung Function and Heterogeneity of Specific Gas Volume in Healthy and Emphysematous Subjects. Eur. Respir. J. 41, 1179–1188. 10.1183/09031936.00050112 22878884

[B3] Arora N. S. Rochester D. F. (1982). Respiratory Muscle Strength and Maximal Voluntary Ventilation in Undernourished Patients. Am. Rev. Respir. Dis. 126, 5–8. 10.1164/arrd.1982.126.1.5 7091909

[B4] Bhatt S. P. Sieren J. C. Dransfield M. T. Washko G. R. Newell J. D. Jr. Stinson D. S. (2014). Comparison of Spirometric Thresholds in Diagnosing Smoking-Related Airflow Obstruction. Thorax 69, 409–414. 10.1136/thoraxjnl-2012-202810 23525095 PMC4146523

[B5] Bodduluri S. Bhatt S. P. Hoffman E. A. Newell J. D. Jr. Martinez C. H. Dransfield M. T. (2017). Biomechanical CT Metrics Are Associated with Patient Outcomes in COPD. Thorax 72, 409–414. 10.1136/thoraxjnl-2016-209544 28044005 PMC5526353

[B6] Bodduluri S. Reinhardt J. M. Hoffman E. A. Newell J. D. Jr. Bhatt S. P. (2018). Recent Advances in Computed Tomography Imaging in Chronic Obstructive Pulmonary Disease. Ann. ATS 15, 281–289. 10.1513/annalsats.201705-377fr PMC5880521 28812906

[B7] Boedeker K. L. Mcnitt-Gray M. F. Rogers S. R. Truong D. A. Brown M. S. Gjertson D. W. (2004). Emphysema: Effect of Reconstruction Algorithm on CT Imaging Measures. Radiology 232, 295–301. 10.1148/radiol.2321030383 15220511

[B8] Borg G. A. (1982). Psychophysical Bases of Perceived Exertion. Med. Sci. Sports Exerc. 14, 337. 7154893

[B9] Braun S. R. Keim N. L. Dixon R. M. Clagnaz P. Anderegg A. Shrago E. S. (1984). The Prevalence and Determinants of Nutritional Changes in Chronic Obstructive Pulmonary Disease. Chest 86, 558–563. 10.1378/chest.86.4.558 6478894

[B10] Burkes R. M. Panos R. J. Borchers M. T. (2021). How Might Endotyping Guide Chronic Obstructive Pulmonary Disease Treatment? Current Understanding, Knowledge Gaps and Future Research Needs. Curr. Opin. Pulm. Med. 27, 120–124. 10.1097/mcp.0000000000000751 33394748 PMC8480198

[B11] Ding K. Cao K. Fuld M. K. Du K. Christensen G. E. Hoffman E. A. (2012). Comparison of Image Registration Based Measures of Regional Lung Ventilation from Dynamic Spiral CT with Xe-CT. Med. Phys. 39, 5084–5098. 10.1118/1.4736808 22894434 PMC3416881

[B12] Fleming W. (2012). Functions of Several Variables. New York, Heidelberg, Berlin: Springer Science & Business Media.

[B13] Garudadri S. Woodruff P. G. (2018). Targeting Chronic Obstructive Pulmonary Disease Phenotypes, Endotypes, and Biomarkers. Ann. ATS 15, S234–S238. 10.1513/annalsats.201808-533mg PMC6321999 30758998

[B14] Gattinoni L. Pelosi P. Crotti S. Valenza F. (1995). Effects of Positive End-Expiratory Pressure on Regional Distribution of Tidal Volume and Recruitment in Adult Respiratory Distress Syndrome. Am. J. Respir. Crit. Care Med. 151, 1807–1814. 10.1164/ajrccm.151.6.7767524 7767524

[B15] Graham B. L. Steenbruggen I. Miller M. R. Barjaktarevic I. Z. Cooper B. G. Hall G. L. (2019). Standardization of Spirometry 2019 Update. An Official American Thoracic Society and European Respiratory Society Technical Statement. Am. J. Respir. Crit. Care Med. 200, e70–e88. 10.1164/rccm.201908-1590st 31613151 PMC6794117

[B16] Han M. K. Agusti A. Calverley P. M. Celli B. R. Criner G. Curtis J. L. (2010). Chronic Obstructive Pulmonary Disease Phenotypes: The Future of COPD. Am. J. Respir. Crit. Care Med. 182, 598–604. 10.1164/rccm.200912-1843cc 20522794 PMC6850732

[B39] Heinrich M. P. Jenkinson M. Brady M. Schnabel J. A. (2013). MRF-Based Deformable Registration and Ventilation Estimation of Lung CT. IEEE Transactions on Medical Imaging 32, 1239–1248. 10.1109/TMI.2013.2246577 23475350

[B17] Hogg J. C. Macklem P. T. Thurlbeck W. M. (1969). The Resistance of Collateral Channels in Excised Human Lungs. J. Clin. Invest. 48, 421–431. 10.1172/jci105999 5773080 PMC535706

[B18] Hunter A. M. Carey M. A. Larsh H. W. (1981). The Nutritional Status of Patients with Chronic Obstructive Pulmonary Disease. Am. Rev. Respir. Dis. 124, 376–381. 10.1164/arrd.1981.124.4.376 6794394

[B19] Kauczor H.-U. Wielpütz M. O. Jobst B. J. Weinheimer O. Gompelmann D. Herth F. J. F. (2019). Computed Tomography Imaging for Novel Therapies of Chronic Obstructive Pulmonary Disease. J. Thorac. Imaging 34, 202–213. 10.1097/rti.0000000000000378 30550404

[B20] Laboratories A. T. S. C. O. P. S. F. C. P. F. (2002). ATS Statement: Guidelines for the Six-Minute Walk Test. Am. J. Respir. Crit. Care Med. 166, 111–117. 10.1164/ajrccm.166.1.at1102 12091180

[B21] Lange P. Halpin D. M. O'donnell D. E. Macnee W. (2016). Diagnosis, Assessment, and Phenotyping of COPD: beyond FEV₁. Int. J. Chron. Obstruct Pulmon Dis. 11, 3–12. 10.2147/COPD.S85976 26937185 PMC4765947

[B22] Macklem P. T. Mead J. (1967). The Physiological Basis of Common Pulmonary Function Tests. Arch. Environ. Health Int. J. 14, 5–9. 10.1080/00039896.1967.10664685 6017097

[B23] Manian P. (2019). Chronic Obstructive Pulmonary Disease Classification, Phenotypes and Risk Assessment. J. Thorac. Dis. 11, S1761–S1766. 10.21037/jtd.2019.05.10 31632753 PMC6783724

[B24] Martin R. J. Bel E. H. Pavord I. D. Price D. Reddel H. K. (2019). Defining Severe Obstructive Lung Disease in the Biologic Era: an Endotype-Based Approach. Eur. Respir. J. 54, 2019. 10.1183/13993003.00108-2019 PMC6917363 31515397

[B25] Otis A. B. Mckerrow C. B. Bartlett R. A. Mead J. Mcilroy M. B. Selverstone N. J. (1956). Mechanical Factors in Distribution of Pulmonary Ventilation. J. Appl. Physiol. 8, 427–443. 10.1152/jappl.1956.8.4.427 13286206

[B26] Quanjer P. H. Stanojevic S. Cole T. J. Baur X. Hall G. L. Culver B. H. (2012). Multi-ethnic Reference Values for Spirometry for the 3-95-yr Age Range: the Global Lung Function 2012 Equations. Eur. Respir. J. 40, 1324–1343. 10.1183/09031936.00080312 22743675 PMC3786581

[B27] Reinhardt J. M. Ding K. Cao K. Christensen G. E. Hoffman E. A. Bodas S. V. (2008). Registration-based Estimates of Local Lung Tissue Expansion Compared to Xenon CT Measures of Specific Ventilation. Med. Image Anal. 12, 752–763. 10.1016/j.media.2008.03.007 18501665 PMC2692217

[B28] Sahebjami H. Macgee J. (1983). Changes in Connective Tissue Composition of the Lung in Starvation and Refeeding. Am. Rev. Respir. Dis. 128, 644–647. 10.1164/arrd.1983.128.4.644 6625342

[B29] Sahebjami H. Sathianpitayakul E. (2000). Influence of Body Weight on the Severity of Dyspnea in Chronic Obstructive Pulmonary Disease. Am. J. Respir. Crit. Care Med. 161, 886–890. 10.1164/ajrccm.161.3.9905023 10712338

[B30] Segal L. N. Martinez F. J. (2018). Chronic Obstructive Pulmonary Disease Subpopulations and Phenotyping. J. Allergy Clin. Immunol. 141, 1961–1971. 10.1016/j.jaci.2018.02.035 29884286 PMC5996762

[B31] Simon B. A. (2000). Non-invasive Imaging of Regional Lung Function Using X-ray Computed Tomography. J. Clin. Monit. Comput. 16, 433–442. 10.1023/a:1011444826908 12580227

[B32] Simon B. A. (2005). Regional Ventilation and Lung Mechanics Using X-Ray CT1. Acad. Radiol. 12, 1414–1422. 10.1016/j.acra.2005.07.009 16253853

[B33] Stoel B. C. Bode F. Rames A. Soliman S. Reiber J. H. C. Stolk J. (2008). Quality Control in Longitudinal Studies with Computed Tomographic Densitometry of the Lungs. Proc. Am. Thorac. Soc. 5, 929–933. 10.1513/pats.200804-039qc 19056719

[B34] Van Der Molen T. Miravitlles M. Kocks J. W. (2013). COPD Management: Role of Symptom Assessment in Routine Clinical Practice. Int J Chron Obstruct Pulmon Dis 8, 461. 10.2147/copd.s49392 24143085 PMC3798110

[B35] Vandenbergh E. Van De Woestijne K. P. Gyselen A. (1967). Weight Changes in the Terminal Stages of Chronic Obstructive Pulmonary Disease. Relation to Respiratory Function and Prognosis. Am. Rev. Respir. Dis. 95, 556–566. 10.1164/arrd.1967.95.4.556 6021915

[B36] Victorino J. A. Borges J. B. Okamoto V. N. Matos G. F. J. Tucci M. R. Caramez M. P. R. (2004). Imbalances in Regional Lung Ventilation: a Validation Study on Electrical Impedance Tomography. Am. J. Respir. Crit. Care Med. 169, 791–800. 10.1164/rccm.200301-133oc 14693669

[B37] Washko G. R. Parraga G. Coxson H. O. (2012). Quantitative Pulmonary Imaging Using Computed Tomography and Magnetic Resonance Imaging. Respirology 17, 432–444. 10.1111/j.1440-1843.2011.02117.x 22142490 PMC3312990

[B38] Zhang X. Chen H. Gu K. Chen J. Jiang X. (2021). Association of Body Mass Index with Risk of Chronic Obstructive Pulmonary Disease: A Systematic Review and Meta-Analysis. COPD: J. Chronic Obstructive Pulm. Dis. 18, 101–113. 10.1080/15412555.2021.1884213 33590791

