# Supplementary material for: Computed Tomography Registration-Derived Regional Ventilation Indices Compared to Global Lung Function Parameters in Patients With COPD
Source: Front Physiol. 2022 May 26;13:862186. doi: 10.3389/fphys.2022.862186 (PMC9202420; doi:10.3389/fphys.2022.862186)
Supplement: Supplementary file 1 [file DataSheet1.pdf]

Supplementary Material

1 Supplementary Methods

CT image processing

Before aligning the inspiratory and expiratory volumes, the lung parenchyma and trachea were extracted from other tissues, using the "Lung CT Segmenter" in "Chest Imaging Platform" module of 3D Slicer (http://www.slicer.org). This consists in a region growing algorithm (Adams and Bischof, 1994) based on user-provided seeds, followed by a morphological closing and smoothing of the obtained binary masks. As a prerequisite for the utilized registration algorithm, the masked volumes were then processed to have isotropic voxels and identical dimensions. The spacing between the isotropic voxels was 0.625 mm and the in-plane matrix size was kept constant as 512×512 for all the images. After an initial rigid alignment of the two volumes, a deformable image registration was performed by the deedsBCV algorithm [https://github.com/mattiaspaul/deedsBCV] (Heinrich et al., 2013a). Deformable image registration consists in warping one image (moving image, I_{mov}) to morphologically match the other image (fixed image, I_{fix}). The algorithm uses a dense displacement sampling strategy (deeds) to cope with large displacements of small structures. Self-similarity context (SSC) descriptors (Heinrich et al., 2013b) used to calculate the similarity measure (data-attachment term) ensure robustness against image-intensity changes, and pair-wise regularization term inferred on a minimum-spanning-tree model controls the smoothness of the estimated transformation Φ. Here the inspiration image was warped (moving image) to match the expiratory image (fixed image). The resulting deformation field is a matrix containing the displacement vectors that align the corresponding voxels between the fixed and moving images. A median filter with a radius of 2 voxels was applied to the images to remove the effect of acquisition noise.

The following outcome measures were computed:

1) the inspiratory – expiratory x-ray density change in Hounsfield units (dHU) between the fixed (expiration) and warped (inspiration) images, $I_{mov} \rightarrow \hat{I}_{mov}, I_{fix} \rightarrow \hat{I}_{fix}$, calculated as:

$$dHU = I_{fix}^{\wedge} - I_{mov}^{\wedge} \quad , \tag{1}$$

where $I_{mov}^{\wedge}$ stands for the filtered moving (inspiratory) image warped by the estimated transformation Φ, and $I_{fix}^{\wedge}$ for the filtered fixed (expiratory) image. Voxels within the interval -1100 to -500 HU were included in the analysis, thus excluding dense structures such as blood vessels.

2) The specific volume change between fixed and warped images, as defined by Simon et al. (Simon, 2000):

$$dsV = \frac{dV}{V_{exp}} = \frac{1000 * dHU}{I_{fix}^{\wedge} \times (I_{mov}^{\wedge} + 1000)} \tag{2}$$

Where *dsV* is specific volume change; *dV* is the local volume change upon inspiration; V_{exp} is the local gas volume at end-expiration; *dHU* the attenuation change (Ding et al., 2012).

3) The determinant of the Jacobian matrix was calculated from the non-linear transformation function: Φ (Reinhardt et al., 2008). This parameter referred to as the Jacobian (J), expresses the local relative volume change between inspiration and expiration (Fleming, 2012). The Jacobian is independent of the image intensity values and therefore of *dHU*. A Jacobian value greater than 1 means local expansion whereas a $J<1$ indicates local contraction and $J=1$ indicates no volume change.

Scattering in *dHU*, *dsV* and *J* was expressed as the quantile variation coefficient (QVC) defined as interquartile range/median rather than coefficient of variation, given the non-normal distribution of these parameters.

2 Supplementary Tables

Supplemental Table 1. Correlation between the kurtosis of regional lung function parameter distribution and demographic, global lung function, gas exchange exercise capacity, dyspnoea and GOLD classification.

	J-kurtosis		*dHU- kurtosis*		*dsV- kurtosis*	
	R	p	R	p	R	p
**Age yrs**	-0.04	0.685	0.15	0.120	0.03	0.771
**Sex M/F**	0.20	0.042	0.01	0.915	-0.07	0.475
**BMI kg·m^{-2}**	-0.08	0.441	-0.40	0.000	-0.28	0.004
**Smoking pack-yrs**	-0.07	0.498	0.07	0.469	0.12	0.244
**FVC %pred**	0.13	0.187	-0.25	0.012	-0.31	0.002
**FEV1 %pred**	0.09	0.399	-0.48	0.000	-0.46	0.000
**FEV1/FVC %**	0.01	0.901	-0.54	0.000	-0.45	0.000
**FEF25-75 %pred**	0.03	0.790	-0.44	0.000	-0.42	0.000
**TLC %pred**	0.16	0.122	0.26	0.011	0.26	0.011
**RV %pred**	0.05	0.632	0.30	0.003	0.37	0.000
**RV/TLC %**	-0.15	0.132	0.38	0.000	0.44	0.000
**DLCOcorr %pred**	0.22	0.105	-0.46	0.000	-0.39	0.002
**PaO_2 kPa**	0.01	0.901	-0.27	0.009	-0.35	0.001
**$PaCO_2$ kPa**	0.05	0.638	0.15	0.143	0.14	0.185
**SaO_2 %**	0.00	0.993	-0.23	0.025	-0.31	0.002
**6MWD m**	0.17	0.100	-0.27	0.008	-0.34	0.001
**Dyspnoea score**	-0.10	0.485	0.19	0.176	0.28	0.042
**GOLD**	-0.12	0.242	0.41	0.000	0.43	0.000

dHU-kurtosis: density change distribution kurtosis; dsV-kurtosis: specific volume change distribution kurtosis; J-kurtosis: Jacobian determinant kurtosis; R: Spearman correlation coefficient; M/F: male/female; BMI: body mass index; FEV1: forced expiratory volume in one second; FVC: forced vital capacity; FEF25-75: forced mid-expiratory flow; RV: plethysmographic residual volume; TLC: plethsymographic total lung capacity; DLCOcorr; diffusing capacity or carbon monoxide, corrected for haemoglobin value; PaO_2: arterial O_2 pressure; $PaCO_2$: arterial CO_2 pressure; SaO_2: arterial O_2 saturation; 6MWD: 6-minute walk distance; GOLD: Global initiative for Chronic Obstructive Lung Disease class.

Supplemental Table 2. Correlation between the skewness of regional lung function parameter distribution and demographic, global lung function, gas exchange exercise capacity, dyspnoea and GOLD classification.

	J-skewness		*dHU- skewness*		*dsV- skewness*	
	R	p	R	p	R	p
**Age yrs**	-0.02	0.873	0.13	0.206	0.11	0.270
**Sex M/F**	0.22	0.030	0.20	0.040	0.05	0.634
**BMI kg·m^{-2}**	-0.10	0.336	-0.36	0.000	-0.32	0.001
**Smoking pack-yrs**	-0.01	0.891	-0.02	0.871	0.10	0.336
**FVC %pred**	0.12	0.235	0.05	0.601	-0.22	0.026
**FEV1 %pred**	0.11	0.294	-0.23	0.021	-0.46	0.000
**FEV1/FVC %**	0.05	0.588	-0.44	0.000	-0.54	0.000
**FEF25-75 %pred**	0.06	0.532	-0.28	0.005	-0.46	0.000
**TLC %pred**	0.09	0.407	0.21	0.039	0.30	0.003
**RV %pred**	-0.01	0.953	0.17	0.105	0.35	0.001
**RV/TLC %**	-0.18	0.082	0.06	0.532	0.35	0.000
**DLCOcorr %pred**	0.27	0.044	-0.21	0.109	-0.35	0.007
**PaO_2 kPa**	0.07	0.521	-0.04	0.668	-0.31	0.002
**$PaCO_2$ kPa**	0.00	0.975	0.03	0.740	0.10	0.350
**SaO_2 %**	0.03	0.767	-0.04	0.681	-0.29	0.004
**6MWD m**	0.17	0.092	-0.07	0.519	-0.28	0.006
**Dyspnoea score**	-0.14	0.333	0.04	0.792	0.25	0.079
**GOLD**	-0.12	0.236	0.17	0.079	0.41	0.000

dHU-kurtosis: density change distribution kurtosis; dsV-kurtosis: specific volume change distribution kurtosis; J-kurtosis: Jacobian determinant kurtosis; R: Spearman correlation coefficient; M/F: male/female; BMI: body mass index; FEV1: forced expiratory volume in one second; FVC: forced vital capacity; FEF25-75: forced mid-expiratory flow; RV: plethysmographic residual volume; TLC: plethsymographic total lung capacity; DLCOcorr; diffusing capacity or carbon monoxide, corrected for haemoglobin value; PaO_2: arterial O_2 pressure; $PaCO_2$: arterial CO_2 pressure; SaO_2: arterial O_2 saturation; 6MWD: 6-minute walk distance; GOLD: Global initiative for Chronic Obstructive Lung Disease class.

2.1 Supplementary Figures

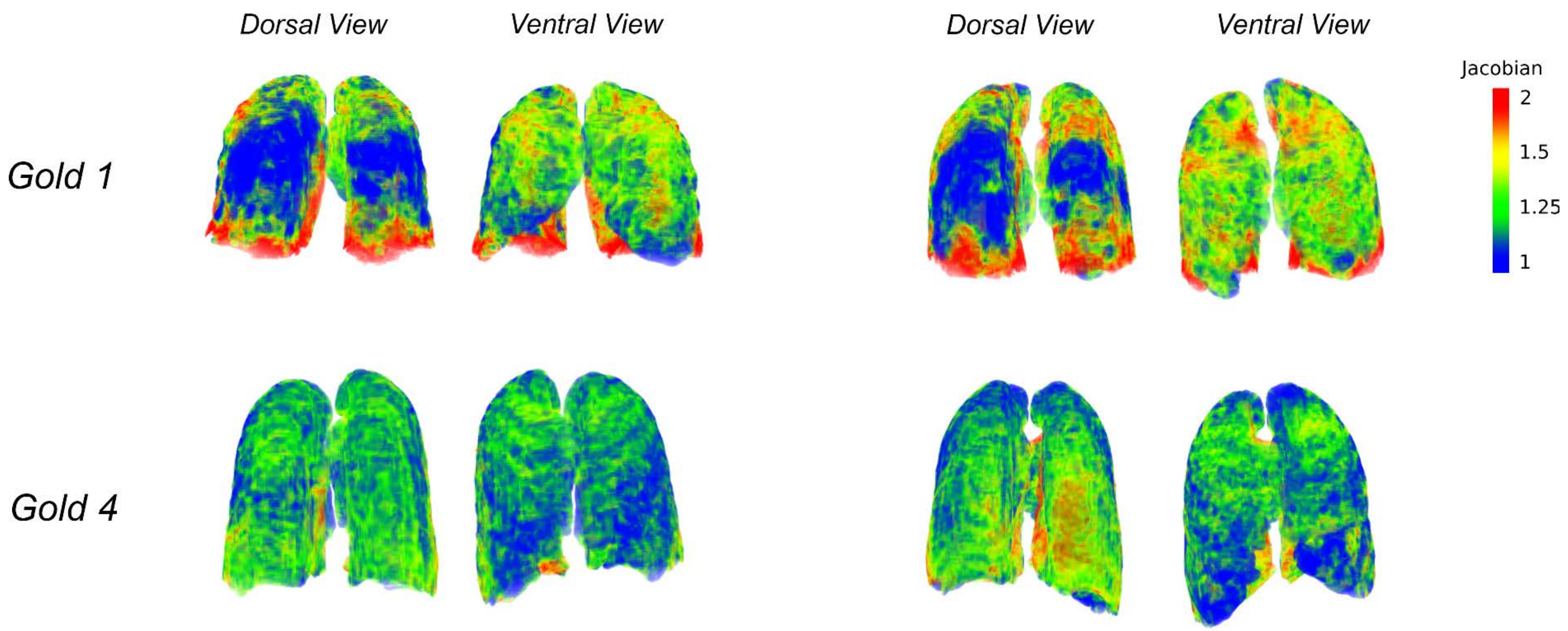

Supplemental Figure 1. Dorsal and ventral views of the 3D rendering of the Jacobian determinant in two GOLD stage 1 and two GOLD stage 4 patients. Note the low values of the Jacobian in the dependent dorsal regions in the GOLD 1 patients, while the dorsal – ventral regional differences were reduced in the GOLD 4 patients.

References

Adams, R., and Bischof, L. (1994). Seeded region growing. *Pattern Analysis and Machine Intelligence, IEEE Transactions on* 16**,** 641-647.

Ding, K., Cao, K., Fuld, M.K., Du, K., Christensen, G.E., Hoffman, E.A., and Reinhardt, J.M. (2012). Comparison of image registration based measures of regional lung ventilation from dynamic spiral CT with Xe-CT. *Medical Physics* 39**,** 5084-5098.

Fleming, W. (2012). *Functions of several variables.* Springer Science & Business Media.

Heinrich, M.P., Jenkinson, M., Brady, M., and Schnabel, J.A. (2013a). MRF-based deformable registration and ventilation estimation of lung CT. *IEEE Transactions on Medical Imaging* 32**,** 1239-1248.

Heinrich MP, Jenkinson M, Papież BW, Brady SM, Schnabel JA. Towards realtime multimodal fusion for image-guided interventions using self-similarities. In: International Conference on Medical Image Computing and Computer-Assisted Intervention - *MICCAI* (2013b) pp. 187-194. Springer, Berlin, Heidelberg.

Reinhardt, J.M., Ding, K., Cao, K., Christensen, G.E., Hoffman, E.A., and Bodas, S.V. (2008). Registration-based estimates of local lung tissue expansion compared to xenon CT measures of specific ventilation. *Med Image Anal* 12**,** 752-763.

Simon, B.A. (2000). Non-invasive imaging of regional lung function using x-ray computed tomography. *J Clin Monit Comput* 16**,** 433-442.
